# Et Tu, B12? Cobalamin Deficiency Masquerading As Pseudo-Thrombotic Microangiopathy

**DOI:** 10.7759/cureus.9097

**Published:** 2020-07-09

**Authors:** Swathi Rao, Daniel Colon Hidalgo, Jorge A Doria Medina Sanchez, Deyger Navarrete, Stephanie Berg

**Affiliations:** 1 Internal Medicine, MacNeal Hospital, Berwyn, USA; 2 Nephrology, Los Angeles County+USC Medical Center, Los Angeles, USA; 3 Hematology and Oncology, Loyola University Medical Center, Maywood, USA

**Keywords:** megaloblastic anemia, non-immune hemolytic anemia, thrombotic microangiopathy, pseudo-thrombotic microangiopathy, vitamin b12 deficiency, reticulocytopenia, intramedullary hemolysis, pancytopenia, moschcowitz syndrome, vitamin b12

## Abstract

Vitamin B12 deficiency is classically associated with megaloblastic anemia. Possible cobalamin deficiency is not investigated once hemolysis is seen. Around 2.5% of cases can present as pseudo-thrombotic microangiopathy (TMA). A swift identification of this means the difference between an easy solution and a protracted one for the patient. A 74-year-old man with no past medical history presented with exertional dyspnea, fatigue, and increasing anorexia over two weeks. Physical examination including a neurological examination was normal. Laboratory tests revealed pancytopenia, unconjugated hyperbilirubinemia, elevated LDH (lactate dehydrogenase), low haptoglobin, and fragmented red blood cells (RBCs) on the peripheral smear, but normal FDP (fibrinogen degradation product) and fibrinogen. The absolute reticulocyte count was reduced as opposed to the expected elevation. Vitamin B12 levels were undetectable, and severe cobalamin deficiency from pernicious anemia was found to be the paramount etiology. Cobalamin deficiency causing pseudo-TMA baffles most physicians. Advanced pernicious anemia is thought to cause intramedullary hemolysis, resulting in peripheral pancytopenia. The fragile RBCs are easily sheared, producing schistocytosis without platelet microthrombi. In contrast to hemolytic anemias, reticulocyte count is low given the unavailability of B12 for erythropoiesis. Reticulocytopenia is a universal finding in cases of pseudo-TMA. Around 38.8% of cases with pseudo-TMA are misdiagnosed as thrombotic thrombocytopenic purpura and treated with plasma product therapy. Keeping an eye out for reticulocytopenia in cases of hemolysis could mean a world of difference for the patient.

## Introduction

Vitamin B12 deficiency (cobalamin) is classically associated with megaloblastic anemia. The concern for cobalamin deficiency is not typically investigated once hemolysis is seen. Ten percent of B12 deficiencies present with pancytopenia or hemolysis [1]. Even rarer are cases mimicking a picture of thrombotic microangiopathy (TMA), which is only around 2.5%. The swift identification of this association is imperative in developing an appropriate differential for the diagnosis of cobalamin deficiency and its hematological associations.

## Case presentation

A 74-year-old man with no significant past medical history presented to the emergency department with a constellation of non-specific symptoms. Over the previous two weeks, he had exertional dyspnea (NYHA [New York Heart Association] class I-II) associated with a diffuse uncomfortable feeling in his chest, generalized weakness, and increasing anorexia. In the last four years, he was admitted to three other hospitals for similar complaints and had undergone extensive cardiac testing that was unrevealing to the etiology of his symptoms.

At admission, his vitals were normal. A physical examination including a neurological examination was normal.

**Figure 1:**
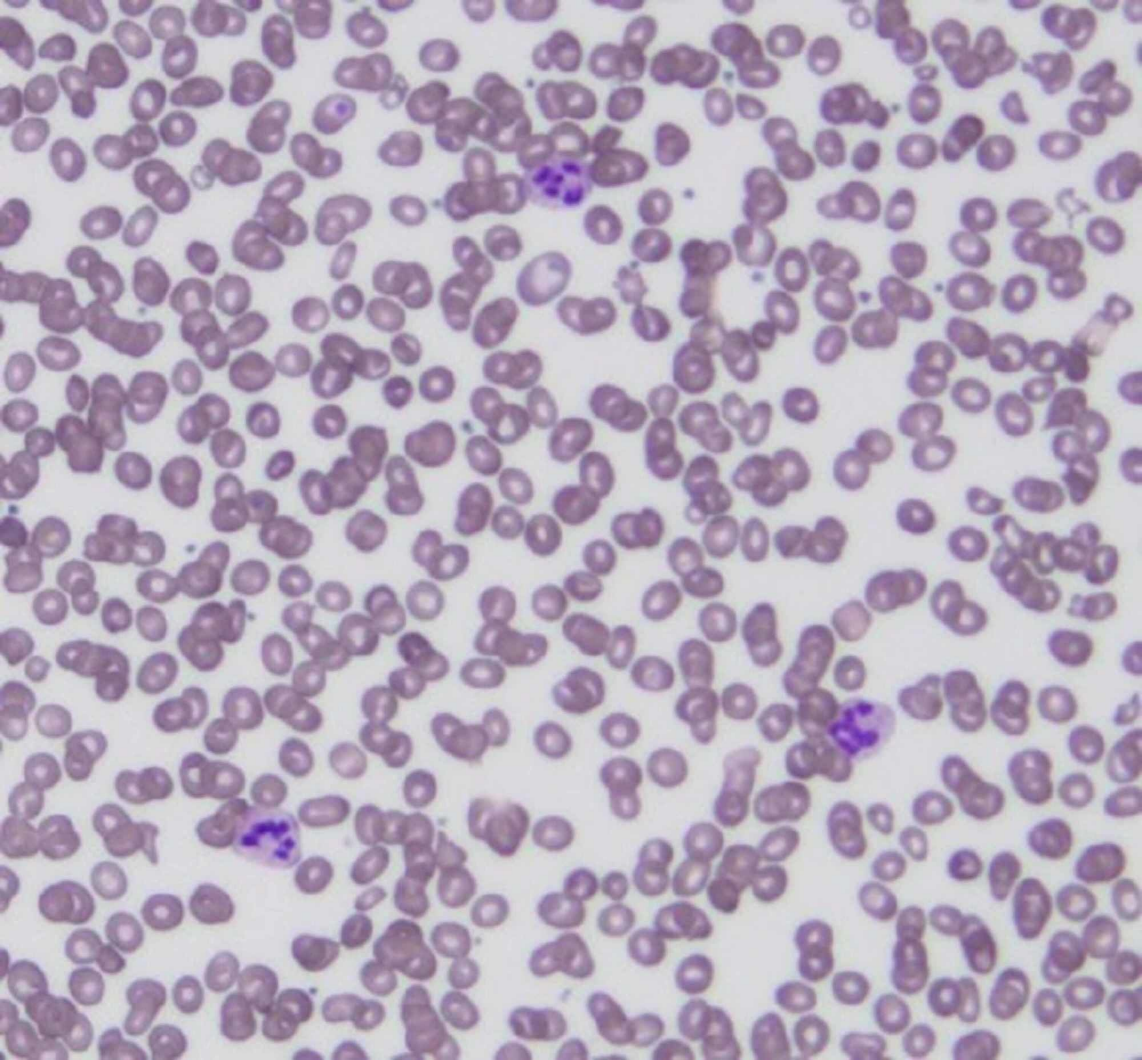
Patient's peripheral smear showing multiple hypersegmented neutrophils.

Laboratory testing revealed pancytopenia (hemoglobin level of 8.9 g/dL, hematocrit of 24.1%, white blood cell count of 3.8 K/uL, platelet count of 68 K/uL) with unconjugated hyperbilirubinemia (total bilirubin 2.4 mg/dL, indirect bilirubin 1.5 mg/dL), elevated lactate dehydrogenase (LDH) (621 IU/L), and low haptoglobin (<8 mg/dL). Fragmented red blood cells (RBCs) were found on the peripheral smear (Figure 1). With growing concern for TMA, fibrinogen and fibrin degradation products were tested and were found to be within the normal range, 232.7 mg/dL and <10 mcg/dL, respectively. Hemolytic anemia was considered, but the absolute reticulocyte count was reduced (0.7/mm^3^) with a low reticulocyte index (0.37%), as opposed to the expected elevation, suggesting hypoproliferation. The mean corpuscular volume (MCV) suggested macrocytosis (128 fL), and a more detailed workup for causes of anemia was done. Iron studies and folate levels were normal: iron 129 mcg/dL, iron binding 214 mcg/dL, iron saturation 60.3%, and folate of 12.53 ng/dL. The vitamin B12 level was zero, with positive anti-parietal cell and anti-intrinsic factor antibodies, and severe cobalamin deficiency from pernicious anemia was found to be the paramount etiology.

## Discussion

Cobalamin is a co-factor vital to DNA/RNA synthesis and fatty acid metabolism. The most common causes of deficiency are inadequate dietary intake (in children) and pernicious anemia (in adults). B12 deficiency is a reversible cause of “bone-marrow failure” [2]. Deficiency of the vitamin has been known to cause a syndrome with pancytopenia, megaloblastic anemia, hypersegmented neutrophils (explained by dysfunctional intramedullary hematopoiesis), and dorsal column dysfunction (explained by dysfunctional myelin synthesis) since time immemorial. Cobalamin deficiency resulting in hemolysis is very rare and is seen in only 10% of deficiencies [1]. Out of those, B12 deficiency causing pseudo-TMA (Moschcowitz syndrome) is even rarer and is seen in only about 2.5% cases [3].

In order to understand pseudo-TMA, we have to first define TMA. TMA is a pathophysiological banner that includes multiple disease processes that ultimately cause intravascular platelet aggregates or microthrombi. This is therefore an occlusive micro or macrovascular disease that results in shearing of RBCs over the microthrombi as they circulate. Clinically, this manifests as microangiopathic hemolytic anemia and consumptive thrombocytopenia (MAHAT) [4]. Thrombotic thrombocytopenic purpura (TTP) is a form of TMA resulting from reduced ADAMTS-13 (a disintegrin and metalloprotease with thrombospondin type 1 motif, member 13) activity. It leads to encephalopathy and renal failure and is a medical emergency.

On the other hand, advanced pernicious anemia is thought to cause hemolysis intramedullary, resulting in peripheral pancytopenia [5-8]. The RBCs being fragile are easily sheared, producing schistocytosis even in the absence of platelet microthrombi. This would be a “pseudo” TMA that clinically looks the same but in reality is completely different. In stark contrast to regular hemolytic anemias, the reticulocyte count here is low, given the unavailability of B12 for the expected compensatory increase in erythropoiesis. A comprehensive literature search by Tran and Tran found reticulocytopenia to be a universal finding in recognized cases of pseudo-TMA [6].

**Figure 2:**
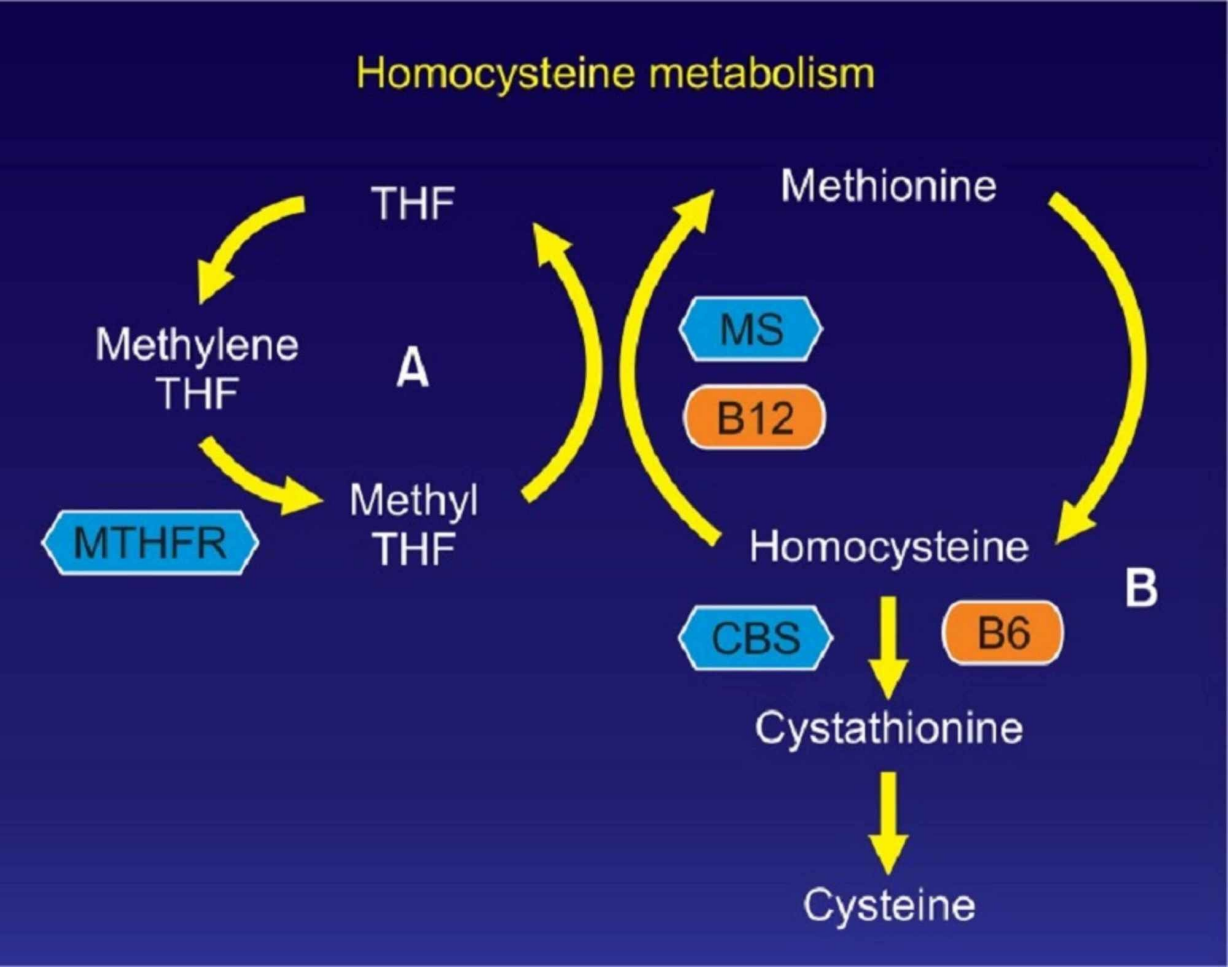
Interdependence of homocysteine and B12. Image taken from Park and Chang [11]. THF, tetrahydrofolate; MTHFR, methylenetetrahydrofolate reductase; MS, methionine synthase; CBS, cystathionine β synthase; B12, vitamin B12; B6, vitamin B6

Another mechanism of hemolysis is thought to stem from hyperhomocysteinemia present in cobalamin deficiency. Without B12, homocysteine cannot be methylated and recycled into methionine, resulting in its accumulation (Figure 2). Homocysteine has been proven a hemolytic toxin in vitro [7]. This cytotoxic effect is estimated to be from its pro-oxidant nature. Homocysteine has been shown to down-regulate the activity of glutathione peroxidase [1], leading to accumulation of reactive oxygen species intracellularly and red cell destruction [8,9]. It is also thought that damage to endothelial cells from homocysteine-induced oxidative stress causes microangiopathy and platelet microthrombi. This results in shearing of the passing RBCs and schistocytosis [3,6,7,10].

Cobalamin deficiency causing hemolysis is a difficult diagnosis to make. Our patient presented with anemia, thrombocytopenia, unconjugated hyperbilirubinemia, elevated LDH, low haptoglobin, and fragmented RBCs on the peripheral smear. These are all red flags for TMA. A comprehensive literature search by Tun et al. in 2017 found that around 38.8% of cases with pseudo-TMA resulting from cobalamin deficiency were misdiagnosed as TTP and treated with plasma product therapy. It took a median duration of about 2 weeks before the diagnosis was corrected [12,13].

Further complicating the diagnosis is the absence of any neurological deficits in our patient. A review of the literature revealed that this has been observed before. B12 is a reversible cause of demyelination of the nervous system. It presents with a wide variety of neuropsychiatric manifestations. The most common findings include myelopathy, peripheral neuropathy, optic neuropathy, altered mental status, and dementia. The presence of such deficits is seen only in about 40% of cases of cobalamin deficiency. Therefore, the absence of neurological deficits does not rule in or rule out cobalamin deficiency. Furthermore, for unclear reasons, an inverse relationship has been found between the severity of anemia and neurological damage [2].

Some other findings can also help in differentiating TTP/TMA from pseudo-TMA. High levels of LDH are not to be expected in TTP/TMA, given that LDH can only come from nucleated cells [3]. Hemolysis of mature RBCs in the peripheral vasculature (as seen in TMA) would hence not release LDH into circulation. The presence of macrocytosis is another clue toward pseudo-TMA. This finding, however, can be tainted by the presence of schistocytosis in severe cobalamin deficiency, falsely reducing the MCV [7]. Pernicious anemia is also known to cause autoimmune hemolytic anemia that would result in Coombs-positive microcytic anemia. These features, although logical, are not found in every case, hence being less reliable than reticulocytopenia.

Why have concern over false diagnosis of TTP? The treatment for B12 deficiency induced hemolysis is simply parenteral replacement of B12. In reported cases worldwide, a complete resolution of the findings and symptoms were seen with this simple measure [5,10]. In contrast, TTP is a medical emergency. Patients falsely diagnosed with TTP were unnecessarily triaged to a higher center, receiving aggressive treatments with expensive plasma products and extended intensive care unit stays [12].

## Conclusions

Hemolysis and pseudo-TMA from B12 deficiency is a known but not a well-recognized phenomenon. The presentation could be highly misleading, playing havoc on the diagnostician’s mind. In the face of a case that could so easily be TTP or true hemolysis, keeping an eye out for reticulocytopenia could represent the difference between an easy solution and an unnecessary and aggressive one for the patient.
